# Separation of aligning and leveling stages to control mandibular incisor inclination: A randomized clinical trial

**DOI:** 10.1590/2177-6709.26.2.e2119378.oar

**Published:** 2021-04-30

**Authors:** Pornpat THEERASOPON, Steven J. LINDAUER, Chairat CHAROEMRATROTE

**Affiliations:** 1 University of Phayao, School of Dentistry, Department of Orthodontics (Phayao, Thailand).; 2 Virginia Commonwealth University, School of Dentistry, Department of Orthodontics (Richmond/VA, USA).; 3 Prince of Songkla University, Faculty of Dentistry, Department of Preventive Dentistry (Songkhla, Thailand).

**Keywords:** Leveling and alignment, Mandibular incisor proclination, Non-extraction orthodontic treatment

## Abstract

**Objective:**

To determine whether separating the alignment and leveling phases can reduce proclination of the mandibular incisors.

**Methods:**

Eligibility criteria included Class I subjects with an irregularity index of 3-5 mm, 3-4 mm curve-of-Spee (COS), and non-extraction treatment. Thirty adults were randomly allocated into two groups: (1) Control group was leveled and aligned simultaneously with flat archwires progressively to 0.016x0.022-in stainless-steel; (2) Experimental group was aligned first with 0.014-in-superelastic NiTi with mild accentuated COS, then leveled using 0.016x0.022-in beta-titanium accentuated COS archwires and gradually reduced the curve until flat. Mandibular incisor position and inclination were evaluated by cephalometric analysis. COS and irregularity index were evaluated in study models. Assessment was conducted twice after 0.016-in NiTi and after 0.016x0.022-in stainless-steel archwire placements. Dental changes from cephalograms and models were compared within group using paired *t*-test and between groups using independent *t*-test.

**Results:**

Control group: Round-wire-phase, mandibular incisors tipped labially (4.38° and 1 mm) with intrusion (-1.13 mm); Rectangular-wire-phase, mandibular incisors further intruded and proclined (-0.63 mm and 1.38°). Experimental group: During aligning with round accentuated COS archwires, mandibular incisors tipped very slightly labially (0.75° and 0.50 mm) with no significant intrusion; during leveling with rectangular archwires, incisors majorly intruded (1.75 mm) with slight proclination (1.81°). The experimental group had significant less incisor proclination (control: 5.76°, experimental: 2.56°) with more incisor intrusion (control: -1.75 mm, experimental: -2.13 mm). The COS in experimental group showed significant greater reduction (-2.88 mm) than that of the control group (-1.69 mm).

**Conclusion:**

In control group, mandibular incisor proclination was markedly observed in round archwires, with further proclination caused by rectangular archwires. In experimental group, minimal proclination was exhibited when accentuated COS round archwires were used for aligning. Leveling with rectangular archwires caused less proclination with more COS reduction.

## RESUMO

**Objetivo:**

Avaliar se separar os estágios de alinhamento e nivelamento reduz a projeção dos incisivos inferiores.

**Métodos:**

Os critérios de inclusão foram indivíduos Classe I com índice de irregularidade de 3-5mm, Curva de Spee (CS) de 3-4mm e tratamento sem extrações. Trinta adultos foram alocados aleatoriamente em dois grupos: o Grupo Controle (1) foi simultaneamente alinhado e nivelado com arcos planos progressivamente até atingir o 0,016” x 0,022” de aço inoxidável; o Grupo Experimental (2) foi inicialmente alinhado com arcos superelásticos NiTi 0,014” com CS levemente aumentada, em seguida nivelado com arcos de beta-titânio 0,016” x 0,022” com CS acentuada, que foi gradualmente reduzida até ficar plana. A posição e inclinação dos incisivos inferiores foram avaliadas por meio de análise cefalométrica. A CS e o índice de irregularidade foram avaliados por meio de modelos de estudo. A avaliação foi realizada duas vezes, sendo após a inserção dos arcos NiTi 0,016” e dos arcos 0,016” x 0,022” de aço. As mudanças dentárias visualizadas nos cefalogramas e nos modelos foram comparadas dentro dos grupos utilizando teste *t* pareado e entre os grupos utilizando o teste *t* independente.

**Resultados:**

Grupo Controle: estágio de arco redondo: os incisivos inferiores se inclinaram vestibularmente (4.38° e 1 mm) e intruíram (-1,13mm); estágio de arco retangular: os incisivos inferiores intruíram e se projetaram adicionalmente (-0.63mm e 1,38°). Grupo Experimental: Durante o alinhamento com arcos redondos e CS acentuada, os incisivos inferiores se inclinaram levemente para vestibular (0,75° e 0,50mm), sem intrusão significativa; durante o nivelamento com arcos retangulares, os incisivos, em sua maioria, intruíram (1,75mm), com uma leve projeção (1,81°). O grupo experimental apresentou projeção dos incisivos significativamente menor (controle: 5,76°; experimental: 2,56°), com maior intrusão dos incisivos (controle: -1,75mm; experimental: -2,13mm). A CS no Grupo Experimental apresentou redução significativamente maior (-2,88 mm) do que no grupo controle (-1,69 mm).

**Conclusão:**

No Grupo Controle, foi observada de forma notória a projeção dos incisivos inferiores nos arcos redondos, com projeção adicional causada pelos arcos retangulares. No Grupo Experimental, foi observada uma projeção mínima quando foram utilizados arcos redondos com CS acentuada para alinhamento. O nivelamento com arcos retangulares causou menos projeção com maior redução da CS.

## INTRODUCTION

Alignment and leveling is the first stage of comprehensive orthodontic treatment.^1^ Typically, superelastic archwires, such as nickel-titanium (NiTi) alloys or multi-stranded stainless steel, are used in this stage due to alignment efficiency prior to inserting stiffer archwires.^2^ Results of very effective dental changes early in treatment after alignment and leveling showed that proclination of mandibular incisors was common,^3^ especially in non-extraction orthodontically-treated cases.^4^ ^-^ ^8^ Proclination of mandibular incisors may affect the esthetic outcome, surrounding periodontal tissues,^9^ and treatment stability.^10^

To correct crowded teeth, labial displacement of incisors is expected to gain spaces for tooth alignment.^5^ ^-^ ^8^ ^,^ ^11^ To reduce this flaring, rectangular archwires, which have the ability of torque control, should be placed at the beginning stage.^12^ Unfortunately, the placing of rectangular NiTi archwires with 0.016 x 0.022-in is probably not possible since the dimension of 0.022-in of the archwire cannot be forced into the disordered bracket slots on crowded teeth. Moreover, heavy forces could be expected from rectangular wire deflection.^13^ In order to reduce this force with better torque control, 0.016 x 0.022-inch beta-titanium archwires with multiple loops can be introduced. However, complicated archwire bending, prolonged chair time, and the difficulty of oral hygiene practice must be considered.^14^ Therefore, a conventional sequence making use of the NiTi archwire for aligning is inevitable.

For tooth leveling or curve of Spee (COS) flattening, the placement of plain small round NiTi archwires can cause the incisors to flare up since intrusion forces are generated anterior to the centers of resistance of the incisors.^15^ To counteract this flaring, a rectangular archwire can be applied for leveling after the crowded teeth are aligned.^16^ ^,^ ^17^ The amount of incisor intrusion should be introduced little by little to avoid any heavy force.

Those problems led to the idea of changing the archwire shape to align the teeth with minimal leveling by using small round NiTi archwires with accentuated COS. Leveling can be subsequently approached using 0.016 x 0.022-in beta-titanium archwires with COS, then gradually flattened to generate optimal force.

The objective of the study was to investigate the movement of mandibular incisors focusing on the inclination when conventional round and rectangular archwires were used for aligning and leveling simultaneously, compared to accentuated COS archwires used for alignment followed by rectangular archwires for leveling.

## MATERIAL AND METHODS

### TRIAL DESIGN AND ANY CHANGES AFTER TRIAL COMMENCEMENT

This was a parallel-group prospective, randomized, controlled trial with a 1:1 allocation ratio. No changes were made to the methods during the trial.

### PARTICIPANTS, ELIGIBILITY CRITERIA, AND SETTING

**Figure 1:**
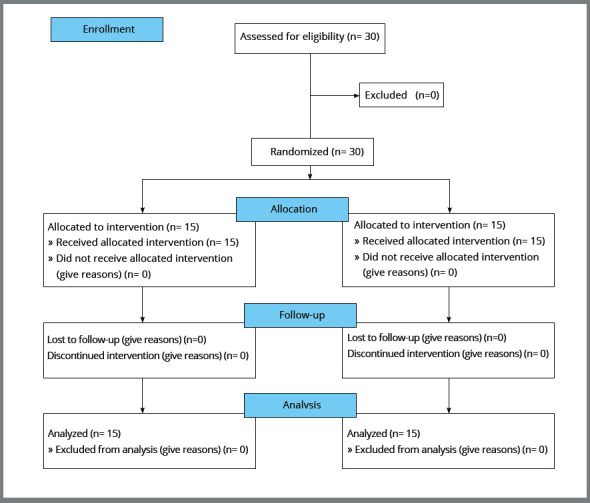
Modified CONSORT 2010 diagram.

The trial was reviewed and approved by the Human Research Ethics Committee of the Faculty of Dentistry, Prince of Songkla University (Project No. EC6101-05-P-HR). The trial was reported according to the Consolidated Standards of Reporting Trials (CONSORT) statement^18^ (Fig 1). Participants were recruited and treated in the orthodontic clinic of a university dental hospital. All subjects were informed of the study objectives and treatment protocol, and informed consent was received from the participants. The subjects were 18-30 years of age. The inclusion criteria included the following: (1) skeletal Class I (ANB angle = 1-5°); (2) all mandibular teeth were present, except third molars; (3) Little’s irregularity index was in the range of 3-5 mm in which each contact was less than 1 mm; (4) the amount of posterior discrepancy was 0-2 mm; (5) COS depth was 3-4 mm; and (6) a non-extraction orthodontic treatment plan was indicated. The exclusion criteria were those individuals who had: (1) attached gingiva less than 1.5 mm, (2) clinical attachment loss more than 4 mm, (3) allergies, (4) systemic diseases, (5) drug use that altered bone metabolism, (6) previous orthodontic treatment, and (7) subjects who failed to attend monthly appointments. 

### INTERVENTIONS

Treatment began with bonding of the maxillary teeth until the mandibular teeth could be bonded using pre-adjusted Roth prescription edgewise brackets (0.018 x 0.025-in, Ormco Mini Diamond^®^, Orange, CA, USA).

The control group was treated with conventional alignment and leveling using 0.014-in and 0.016-in superelastic NiTi archwires (Great Lakes Orthodontics, NY, USA), 0.016 x 0.016-in, and 0.016 x 0.022-in stainless steel (Highland Metals, IN, USA). The experimental group was treated with 0.014-in and 0.016-in superelastic NiTi archwires (Great Lakes Orthodontics, NY, USA) with a shallow reverse COS in an upside-down position (accentuated COS) to minimize altering the original COS during alignment. Then customized 0.016 x 0.016-in stainless steel with a passive COS and 0.016 x 0.022-in beta-titanium archwires (Highland Metals, IN, USA) gradually reduced the curve by 1.5 mm at each appointment until flat. This was followed by 0.016 x 0.022-in straight stainless steel. Appointments were at 3- to 4-week intervals.

Treatment records were obtained at three time points: (1) the initial data were recorded as T_0_; (2) the data at the end of the alignment phase by 0.016-in superelastic NiTi archwires when Little’s irregularity index was near zero and recorded as T_1_; and (3) the data after three months of flat 0.016 x 0.022-in stainless steel archwires in place when the alignment and leveling phase was completed and recorded as T_2_. The records taken at each time point were study models and lateral cephalometric radiographs.

### STUDY MODEL AND CEPHALOMETRIC ANALYSES

**Figure 2:**
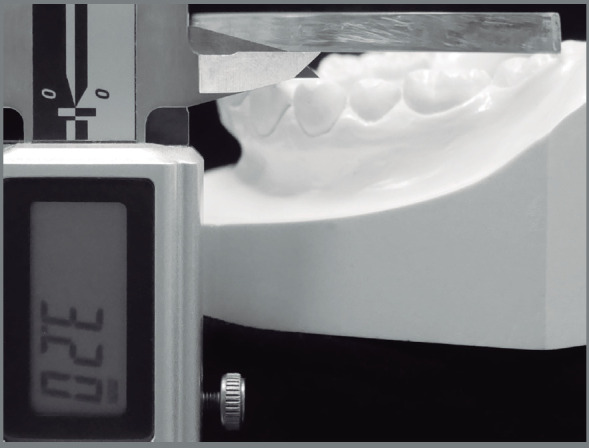
Curve of Spee depth measurement method.

**Figure 3:**
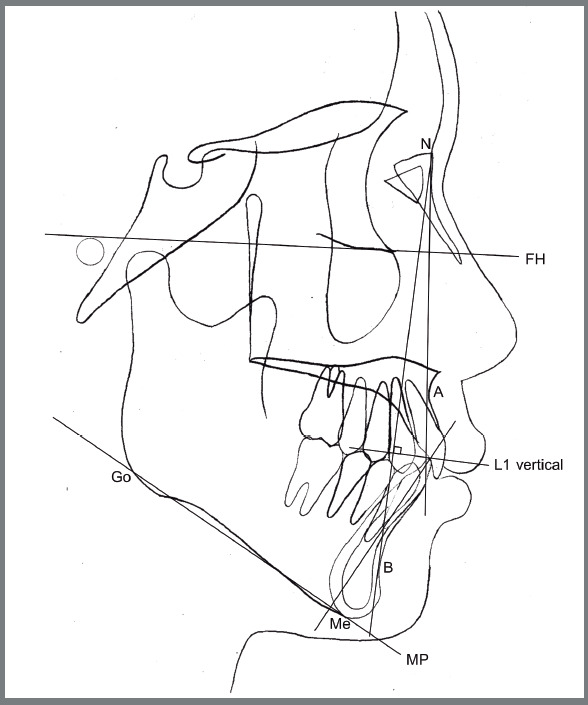
Lateral cephalometric measurements used in this study.

Study models were evaluated using digital Vernier callipers set to zero before the next measurement for the amount of tooth crowding using Little’s irregularity index.^19^ The COS depth was assessed using a clear acrylic plate laid down from the mandibular second molars to the incisors measuring the average depth from the acrylic plate to the most inferior cusp tip of the bilateral premolars (Fig 2). The lateral cephalometric analysis evaluated position and inclination of the mandibular incisors and rotation of the mandibular plane (Fig 3).

### SAMPLE SIZE CALCULATION

The sample size was calculated based on a previous study^20^ with alpha = 0.01 for an independent *t*-test and a statistical power of 90%. The sample size required 13 subjects per group and two additional participants per group were included for possible drop-outs. Thus, the total number of participants in this study was 15 per group.

### INTERIM ANALYSES AND STOPPING GUIDELINES

Not applicable.

### RANDOMIZATION

Patients were divided into two parallel groups using simple randomization by drawing lots. Each participant was numbered for blinding by the first author. The lots were sealed in opaque envelopes for the group assignment. One resident member who was not part of the trial shuffled and opened the envelopes for each participant in a private room to ensure that the operator was blinded.

### BLINDING

Blinding of the patients and operator was not possible; however, assessment was blinded and accomplished by an examiner not involved in patient treatment using lateral cephalometric radiographs and study models that were coded to conceal patient information.

### STATISTICAL ANALYSIS

The data were analyzed using IBM SPSS Statistics 21 software (IBM Software Group, Chicago, IL, USA). Intra-examiner reliability was determined using paired *t*-tests by random selection of 10 cephalometric radiographs and 10 dental models after two weeks. The radiographs were retraced and measurements were repeated.

Means and standard deviations were calculated for all dental casts and lateral cephalometric radiograph parameters at T_0_, T_1,_ and T_2_. The distribution normality of parameters was tested by the Shapiro-Wilk test. Paired *t*-tests were used to analyse differences among T_0_, T_1,_ and T_2_ of the control and experimental groups. Independent *t*-tests were used to analyze differences between the control and experimental groups. A significant difference level of *p* < 0.05 was used for all statistical tests.

## RESULTS

Thirty patients (11 males/19 females) with a mean age of 22.48 years were randomized in a 1:1 ratio into the control and experimental groups. No subjects were lost during the trial. The participant flow followed the modified CONSORT 2010 flow diagram (Fig 1). Thirty subjects were recruited into the trial between October 2017 and April 2018. The first initial records were taken in March 2018 and the final set in January 2019. All 30 subjects were analyzed and none of the subjects missed any timepoints.

Intra-examiner reliability revealed no significant differences between repeated measurements (*p* > 0.05). Table 1 shows the demographic characteristics of the patients in each group. No differences in these factors between the two groups were noted, except the treatment time in the experimental group (35.13 weeks) was significantly longer than the control group (26.63 weeks) (*p* < 0.0001). Group differences of dental changes from the cephalometric analyses and model measurements at all timepoints are shown in Table 2.

**Table 1:** Descriptive statistics for control and experimental groups.

Male:female ratio (%)	Total (n=30)		Control (n=15)		Experiment (n=15)		P-value
	11:19 (36.67%:63.33%)		5:10 (33.33%:66.67%)		6:9 (40.00%:60.00%)		
	mean	SD	mean	SD	mean	SD	
Age (years)	22.48	4.17	22.44	4.90	22.51	3.65	0.968
Overjet	2.85	0.68	3.07	0.62	2.63	0.69	0.083
Overbite	4.32	0.68	4.47	0.83	4.17	0.45	0.230
Irregularity index (mm)	3.75	0.74	3.91	0.74	3.59	0.73	0.235
ANB (degrees)	2.38	0.88	2.40	0.91	2.37	0.88	0.749
Curve of Spee (mm)	3.34	0.47	3.33	0.52	3.44	0.50	0.709
FMA (degrees)	25.88	3.52	24.75	2.93	27.00	3.88	0.284
Treatment time (weeks)	30.88	4.94	26.63	2.13	35.13	2.53	<0.0001*

*Significant difference between groups (p<0.05).

**Table 2:** Dental changes from lateral cephalometric and model analysis.

Variables		Control (n=15)		Experiment (n=15)		P-value
		mean	SD	mean	SD	
L1 to NB (mm)	T_0_	7.38	1.30	8.50	1.31	0.270
	T_1_	8.38	1.30	9.00	1.31	0.652
	T_2_	8.00	1.22	9.25	1.28	0.202
	T_1_-T_0_	1.00	0.00	0.50	0.00	0.655
	T_2_-T_1_	-0.38	0.23	0.13	0.23	<0.0001*
	T_2_-T_0_	0.63	0.23	0.63	0.23	0.215
L1 to NB (degrees)	T_0_	27.38	4.41	33.00	2.00	0.020*
	T_1_	31.75	4.12	33.75	2.17	0.419
	T_2_	33.13	3.87	35.56	1.97	0.297
	T_1_-T_0_	4.38	0.44	0.75	0.38	<0.0001*
	T_2_-T_1_	1.38	0.44	1.81	0.80	0.408
	T_2_-T_0_	5.76	0.89	2.56	0.94	<0.0001*
FMA (degrees)	T_0_	24.75	2.93	27.00	3.88	0.284
	T_1_	25.00	2.84	27.25	3.78	0.271
	T_2_	25.13	2.76	27.25	3.78	0.302
	T_1_-T_0_	0.25	0.42	0.25	0.38	0.709
	T_2_-T_1_	0.17	0.26	0.00	0.00	0.089
	T_2_-T_0_	0.42	0.49	0.25	0.38	0.486
Irregularity index (mm)	T_0_	3.91	0.74	3.59	0.73	0.235
	T_1_	0.21	0.10	0.27	0.15	0.166
	T_2_	0.07	0.14	0.12	0.09	0.262
	T_1_-T_0_	-3.70	0.77	-3.31	0.71	0.161
	T_2_-T_1_	-0.11	0.17	-0.15	0.10	0.694
	T_2_-T_0_	-3.88	0.79	-3.68	0.74	0.155
∆ L1 vertical (mm)	T_1_-T_0_	-1.13	0.44	-0.38	0.23	<0.0001*
	T_2_-T_1_	-0.63	0.23	-1.75	0.38	<0.0001*
	T_2_-T_0_	-1.75	0.60	-2.13	0.35	0.569
∆ COS (mm)	T_1_-T_0_	-1.19	0.37	-0.19	0.37	0.709
	T_2_-T_1_	-0.50	0.00	-2.69	0.37	0.001*
	T_2_-T_0_	-1.69	0.37	-2.88	0.35	<0.0001*

*Significant difference within group and between groups (*p* < 0.05).

In the control group, mandibular incisors were significantly aligned and leveled by round archwires and it was found that the mandibular incisors moved labially 1 mm and were significantly proclined 4.38° (*p*< 0.0001). In the vertical dimension, the mandibular incisors were intruded 1.13 mm (*p*< 0.0001) with COS reduction of 1.19 mm (*p*< 0.0001). After changing to rectangular archwires, the mandibular incisors intruded further by 0.63 mm (*p*< 0.0001) and proclined slightly 1.38° (*p*< 0.0001). Mandibular incisors moved very little lingually without statistical significance. The total COS reduction was 1.69 mm.

In the experimental group, tooth alignment was significantly performed by accentuated COS round archwires. The mandibular incisors moved 0.50 mm labially with 0.75° proclination without statistical significance. For mandibular incisors, no significant intrusion with no significant COS reduction was observed. When leveling by rectangular archwires, the mandibular incisors intruded by 1.75 mm (*p* < 0.0001) with 1.81° proclination (*p* < 0.0001) without significant labial movement (0.13 mm, *p*= 0.170). The COS was reduced by 2.69 mm (*p* < 0.0001).

Crowding was almost completely resolved by 0.014-in and 0.016-in superelastic NiTi archwires in both control and experimental groups, and T_1_ records were taken. At T_1_, the irregularity indices in the control and experimental groups were significant reduced from 3.91 mm and 3.59 mm to 0.21 mm and 0.27 mm, respectively (*p*= 0.166). The mandibular incisors moved labially in the control and experimental groups by 1.00 mm and 0.50 mm, respectively (*p*= 0.655). The increase in proclination (0.75°) in the experimental group was significantly less than in the control group (4.38°) (*p*< 0.0001). At T_1_, the amount of intrusion in the experimental group was -0.38 mm, which was significantly less than the control group (-1.13 mm) (*p*< 0.0001). COS reduction in the experimental group was -0.19 mm and -1.19 mm in the control group, no significant different was detected (*p* = 0.709). 

After the placement of the flat 0.016 x 0.022-in stainless steel archwires, alignment and leveling were considered to be complete, and T_2_ records were taken. At T_2_, the mandibular incisors in the experimental group demonstrated labial movement of 0.63 mm, which was equal to the control group (0.63 mm) (*p*= 0.215). The increase in incisor proclination in the experimental group (2.56°) was significantly less than in the control group (5.76°) (*p*< 0.0001). The incisors had intruded in the experimental group (-2.13 mm) and the intrusion was significantly greater than the control group (-1.75 mm) (*p*= 0.569). The total COS reduction in the experimental group was -2.88 mm and this was significantly greater than in the control group (-1.69 mm) (*p*< 0.0001). Neither group experienced changes in the Frankfort-mandibular plane angle greater than 0.5°. No serious harm such as gingival recession was observed in any of the subjects.

## DISCUSSION

Mandibular incisor proclination is one of the undesirable side effects that occurs during the alignment and leveling stages of orthodontic treatment. Proclination can lead to periodontal damage, including gingival recession and alveolar bone loss.^21^ ^-^ ^25^ To reduce this risk, a light rectangular beta-titanium archwire may be effective in maintaining better torque control^26^ during the leveling stage. In this study, the experimental technique, which controlled the torque during leveling, was more effective in minimizing unwanted incisor flaring, compared to the conventional technique. The rectangular archwire could not be placed, unless accentuated COS round archwire was innovated for aligning without leveling. The final result was significant COS reduction with less proclination. Totally, the COS was reduced by 2.88 mm with 2.56° flaring or 0.89° per mm. In the control group, the COS was reduced by 1.19 mm with 4.38° flaring while using the round archwires and more proclination (1.38°) occurred with a COS reduction of 0.50 mm while using the rectangular archwires. Totally, the COS was reduced by 1.69 mm with 5.76° flaring or 3.44° per mm. Comparing the ratios of COS reduction and proclination within 1 mm intrusion, the separation technique caused less proclination (0.89°) than the conventional procedure.

During alignment with round superelastic archwires, the straight round NiTi archwires in the control group contributed to increased mandibular incisor proclination during alignment and leveling because they applied an intrusive force on the incisors that was facial to the center of resistance.^15^ As a result, the incisors in the control group exhibited the most intrusion and flaring at T_1_. After placing the rectangular archwires, the mandibular incisors continued to procline with some more intrusion. This could imply that after the extreme proclination caused by the round archwire, lingual crown torque, by subsequently placing the rectangular archwire, could not be expected. Moreover, the remaining COS still allowed the intrusion force from the rectangular wire to create more proclination. The rectangular archwires could not reduce the proclination caused from the round archwires because the moment of couple in the bracket slots for torqueing the incisors was much smaller than the moment of intrusive force.

NiTi archwires with preformed accentuated COS matching to an individual’s COS may be an appropriate alternative to achieve alignment in cases where proclination of the mandibular incisors is undesirable or in cases in which a flat COS is not a treatment goal. To achieve leveling in the experimental group, a rectangular beta-titanium archwire was used to gradually reduce the COS while maintaining torque control. Unfortunately, the treatment time in the torque-controlled method was 8.5 weeks longer, which was mainly due to the rectangular archwire stage when the archwire was passively placed followed by gradual reduction of the COS. Incidentally, this technique found significantly less mandibular incisor proclination (control: 5.76°; experimental: 2.56°) with more mandibular incisor intrusion (control: -1.75 mm; experimental: -2.13 mm). Since the amount of intrusion was limited from the gradual COS reduction, the amount of moment of intrusive force would decrease. This allowed the moment of couple in the bracket’s slot to express lingual crown torque. However, the incisors were still proclined, but less than the control group. Thus, this technique could control the mandibular incisor inclination and be beneficial for patients who need deep COS correction whenever incisor proclination is limited.

A comparison between the two groups in the reduction of COS from mandibular incisor intrusion revealed that the control group had the most substantial proclination during the round wire phase (T_1_-T_0_). However, in the experimental group, reduction of the COS occurred mostly during the rectangular wire phase with less proclination. This could imply that the small round archwires could be the archwire of choice for leveling when proclination is allowed.

A previous study by Pandis et al^27^ also reported that proclination of mandibular incisors was the main result of a flattened COS. Their study exhibited a large amount of mandibular incisor proclination (4.70°) after 1 mm leveling of the COS. This was comparable to the control group in this study in the round wire phase (4.38°) that occurred during the T_1_-T_0_ interval of approximately 1 mm mandibular incisor intrusion and COS correction. In the experimental group of this study, less mandibular incisor proclination was found and was attributed to the torque effect of the rectangular archwires. Additionally, AlQabandi et al^28^ leveled mandibular teeth with rectangular archwires; however, they used NiTi archwires, which were not stiff and could not express the required torque. Therefore, the mandibular incisors were still proclined, as observed in the round archwire group. Meling and Odegaard^26^ found that rectangular beta-titanium archwires were 1.6 times stiffer than NiTi archwire. As a result, rectangular beta-titanium archwires may be more suitable for reducing a deepened COS due to their torque effectiveness and the ability to produce the light forces recommended for mandibular incisor intrusion.^12^

This study was a prospective randomized clinical trial that investigated a two-part technique by first incisor aligning and then flattening the COS by controlled mandibular incisor torque, compared to conventional treatment methods. The results from this finding demonstrate two movements, first by round archwires and subsequently, by rectangular archwires. Other studies^5^ ^-^ ^8^ ^,^ ^27^ ^,^ ^29^ reported the results from total treatment with durations of more than a year and included the finishing phase, which may affect the mandibular incisor position from wire bending, torqueing or intermaxillary elastics. 

Proclination is a major concern in this study due to possible risks^9^ ^,^ ^10^. The control group presented statistically significant greater incisor proclination (3.20°) compared to the experimental group. This amount of proclination difference may not be considered to be clinically significant. An additional study of surrounding bone response to these changes would be interesting to confirm whether this amount of proclination is safe for the periodontium. The results of this study can be applied only in non-extraction patients who have similar pre-treatment characteristics of crowding in the mandibular anterior teeth of 3-5 mm and 3-4 mm COS. 

## CONCLUSIONS

In the control group, mandibular incisor proclination was markedly observed in round archwires, with further proclination caused by rectangular archwires. In experimental group, minimal proclination was exhibited when accentuated COS round archwires were used for aligning. Leveling with rectangular archwires caused less proclination with more COS reduction.

## References

[1] Proffit WR, Fields HW, Sarver DM (2013). Contemporary Orthodontics..

[2] Abdelrahman R, Al-Nimri KS, Al Maaitah EF (2015). A clinical comparison of three aligning archwires in terms of alignment efficiency A prospective clinical trial. Angle Orthod.

[3] Baratieri C, Rocha R, Campos C, Menezes L, Ribeiro GLU, Ritter D (2012). Evaluation of the lower incisor inclination during alignment and leveling using superelastic NiTi archwires a laboratory study. Dental Press J Orthod.

[4] Park HK, Sung EH, Cho YS, Mo SS, Chun YS, Lee KJ (2011). 3-D FEA on the intrusion of mandibular anterior segment using orthodontic miniscrews. Korean J Orthod.

[5] Bishara SE, Cummins DM, Zaher AR (1997). Treatment and posttreatment changes in patients with Class II, Division 1 malocclusion after extraction and nonextraction treatment. Am J Orthod Dentofacial Orthop.

[6] Basciftci FA, Usumez S (2003). Effects of extraction and nonextraction treatment on Class I and Class II subjects. Angle Orthod.

[7] Pandis N, Polychronopoulou A, Eliades T (2007). Self-ligating vs conventional brackets in the treatment of mandibular crowding A prospective clinical trial of treatment duration and dental effects. Am J Orthod Dentofacial Orthop.

[8] Yitschaky O, Neuhof MS, Yitschaky M, Zini A (2016). Relationship between dental crowding and mandibular incisor proclination during orthodontic treatment without extraction of permanent mandibular teeth. Angle Orthod.

[9] Choi YJ, Chung CJ, Kim KH (2015). Periodontal consequences of mandibular incisor proclination during presurgical orthodontic treatment in Class III malocclusion patients. Angle Orthod.

[10] Schulhof RJ, Allen RW, Walters RD, Dreskin M (1977). The mandibular dental arch Part I, lower incisor position. Angle Orthod.

[11] Germane N, Lindauer SJ, Rubenstein LK, Revere JH, Isaacson RJ (1991). Increase in arch perimeter due to orthodontic expansion. Am J Orthod Dentofacial Orthop.

[12] Gurgel JA, Pinzan-Vercelino CRM, Powers JM (2011). Mechanical properties of beta-titanium wires. Angle Orthod.

[13] Parvizi F, Rock WP (2003). The load/deflection characteristics of thermally activated orthodontic archwires. Eur J Orthod.

[14] Chambers C, Stewart S, Su B, Sandy J, Ireland A (2013). Prevention and treatment of demineralisation during fixed appliance therapy a review of current methods and future applications. Br Dent J.

[15] Theerasopon P, Kosuwan W, Charoemratrote C (2019). Stress assessment of mandibular incisor intrusion during initial leveling in continuous arch system with different archwire shapes of superelastic nickel-titanium A three-dimensional finite element study. Int J Health Allied Sci.

[16] Archambault A, Major TW, Carey JP, Heo G, Badawi H, Major PW (2010). A comparison of torque expression between stainless steel, titanium molybdenum alloy, and copper nickel titanium wires in metallic self-ligating brackets. Angle Orthod.

[17] Martins RP (2017). Early vertical correction of the deep curve of Spee. Dental Press J Orthod.

[18] Moher D, Hopewell S, Schulz KF, Montori V, Gotzsche PC, Devereaux PJ (2010). CONSORT 2010 Explanation and Elaboration Updated guidelines for reporting parallel group randomised trials. J Clin Epidemiol.

[19] Little RM (1975). The irregularity index a quantitative score of mandibular anterior alignment. Am J Orthod.

[20] Tantikalyaporn C (2014). The effects of lower incisor intrusion in Class II growing patients..

[21] Steiner GG, Pearson JK, Ainamo J (1981). Changes of the marginal periodontium as a result of labial tooth movement in monkeys. J Periodontol.

[22] Engelking G, Zachrisson BU (1982). Effects of incisor repositioning on monkey periodontium after expansion through the cortical plate. Am J Orthod.

[23] Årtun J, Krogstad O (1987). Periodontal status of mandibular incisors following excessive proclination A study in adults with surgically treated mandibular prognathism. Am J Orthod Dentofacial Orthop.

[24] Garlock DT, Buschang PH, Araujo EA, Behrents RG, Kim KB (2016). Evaluation of marginal alveolar bone in the anterior mandible with pretreatment and posttreatment computed tomography in nonextraction patients. Am J Orthod Dentofacial Orthop.

[25] Theerasopon P, Charoemratrote C (2019). Periodontal tissues after level and align lower anterior teeth in non-extraction orthodontic treatment. J Dent Assoc Thai.

[26] Meling TR, Odegaard J (1998). On the variability of cross-sectional dimensions and torsional properties of rectangular nickel-titanium arch wires. Am J Orthod Dentofacial Orthop.

[27] Pandis N, Polychronopoulou A, Sifakakis I, Makou M, Eliades T (2010). Effects of levelling of the curve of Spee on the proclination of mandibular incisors and expansion of dental arches a prospective clinical trial. Aust Orthod J.

[28] AlQabandi AK, Sadowsky C, BeGole EA (1999). A comparison of the effects of rectangular and round arch wires in leveling the curve of Spee. Am J Orthod Dentofacial Orthop.

[29] Erdinc AE, Nanda RS, Isiksal E (2006). Relapse of anterior crowding in patients treated with extraction and nonextraction of premolars. Am J Orthod Dentofacial Orthop.

